# Nature’s grip: Unveiling the architecture and proteomics of the adhesive organ of a hill stream catfish, *Pterygoplichthys disjunctivus*

**DOI:** 10.1371/journal.pone.0333933

**Published:** 2025-10-09

**Authors:** Jitendra Kumar Sharma, Arunima, Usha Kumari, Swati Mittal, Ajay Kumar Mittal

**Affiliations:** 1 Skin Physiology Laboratory, Centre of Advanced Study, Department of Zoology, Institute of Science, Banaras Hindu University, Varanasi, Uttar Pradesh, India; 2 Zoology Section, Mahila Mahavidyalaya, Banaras Hindu University, Varanasi, India; 3 Retired Professor and Former Head of the Department of Zoology, Banaras Hindu University, 9, Mani Nagar, Kandawa, Varanasi, India; Sher-e-Kashmir University of Agricultural Sciences and Technology of Kashmir, INDIA

## Abstract

Light microscopy, scanning electron microscopy, and proteomics analysis using high-resolution accurate mass spectrometry exhibit significant adaptive structural modifications and characteristic proteins in the adhesive organ (AO) of *Pterygoplichthys disjunctivus,* that could assist the adhesion of the fish to the substratum*.* The free surface of the epithelium of AO showed characteristic mound-like tubercles separated by deep furrows. These could enhance surface grip, and assist in creation of vacuum facilitating adhesion effectively. Spine-like unculi on the surface of the epithelium appear to assist clinging of the fish to the substratum as well as to scrape food particles for feeding. Taste buds located on the summit of mound-like tubercles could serve as adaptations to enhance their sensitivity in food selection and in environmental perception. Mucous and serous goblet cell secretions are believed to function as biological adhesives and protective surface lubricants. Proteomic analysis identified 285 differentially expressed proteins in the AO compared to those in the ventral and the dorsal skin. Out of these proteins in the AO, 80 proteins were significantly abundant. These were Periplakin, Desmoplakin, and Filamin A-like (adhesion related proteins); KRT8 and KRT19 (keratinization associated proteins); Myosin-7, Myosin light chain 13, and Tropomyosin 1 (proteins involved in cytoskeletal organization); and Apolipoprotein A1 and Complement Component 9 (proteins related to immune defense). Gene ontology analysis using Enrichr software revealed the enrichment of unique biological functions and pathways. This study provides a comprehensive understanding of the adaptive strategies that enable *P. disjunctivus* to thrive in turbulent hillstream environments. Additionally, the proteomic profile established in this study serves as a foundation for characterizing and comparing skin proteomes across teleost species.

## Introduction

*Pterygoplichthys disjunctivus* [1] (common name: Vermiculated Sailfin Catfish, Armored Sailfin Catfish, Janitor fish, or Crocodile Catfish) belongs to the Family Loricariidae, Order Siluriformes (Taxonomic Serial Number 680351, Integrated Taxonomy Information System, 2025) [2]. Native to the Amazon River Basin in Brazil and Peru, this species has expanded its range across Asia, North America, and South America (Fishbase, 2025) [3]. Adorned with intricate patterns and markings on the body surface, *P. disjunctivus* has attracted the attention of aquarium enthusiasts and thus holds significant commercial value in the ornamental fish trade. It is valued for its hardiness, algae-cleaning ability, and adaptability, which allows it to thrive in both natural and artificial habitats. In general, these fish are nocturnal and typically live in fast-flowing environments, such as hillstreams or torrential rivers, often attached to submerged rocks, wood, aquatic plants, and sediments [4–6]. Fish scrape their food from the surface of the substratum to which they are attached [7].

Skin is the outer covering of the body and forms a critical interface between an animal and its environment. It serves as a barrier to the entry of microorganisms and resists mechanical damage caused by abrasions and shocks encountered in the environment. It is a dynamic organ that plays a pivotal role in species survival and offers protection in several ways [8,9]. A review of the literature reveals a wealth of studies on the structural, functional, and biological properties of fish skin, emphasizing the morphological and surface architectural diversity among different species [10–18]. Several fish species inhabiting hill streams or torrential rivers have developed various morphological adaptations, including adhesive organs, to survive in such rigorous environments. These include *Schizothorax richardsonii* [19], *Gobiesox maeandricus* [20], *Glyptothorax* sp. [21,22], and *Garra* sp. [23–27].

Proteomics involves the analysis and identification of proteins, their functions, and interactions, thereby providing crucial insights into the molecular mechanisms underlying cellular processes. It plays a significant role in understanding how proteins contribute to biological functions and adaptations, making it an essential tool for exploring the physiological and environmental responses of organisms at the molecular level. Mass spectrometry-based proteomic approaches are increasingly being employed to address complex biological questions and are often combined with other omics disciplines, such as genomics, transcriptomics, and metabolomics, to provide a more comprehensive understanding of biological systems [28,29]. This technique has been instrumental in generating comprehensive proteomic reference maps for a range of organisms including human [30] and zebrafish [31]. The existing literature is too extensive to refer to all studies on fish-skin proteomics. Comprehensive reviews include those on *Dicentrarchus labrax* [32], *Cycloterus lumpus* [33,34], *Boleophthalmus pectinirostris* [35], and *Labeo rohita* [36]. Although proteomics has been widely employed in fish skin, studies on adhesive organs in hill-stream fish remain unexplored, leaving a significant gap in our understanding of their molecular composition and functional adaptations. Recent advances in high-resolution mass-spectrometry-based proteomics have significantly enhanced our ability to investigate the molecular foundations of these adaptations.

In this study, we aimed to investigate the structural organization and proteomic profile of the adhesive organ (AO) of *P. disjunctivus* using a combination of light microscopy, scanning electron microscopy (SEM), and high-resolution accurate mass spectrometry (HRAMS). These findings aim to enhance our understanding of the functional biology of *P. disjunctivus* and shed light on how its adhesive strategies have evolved to support ecological success and invasive potential.

## Materials and methods

### Fish collection and acclimatization

**Fig 1:**
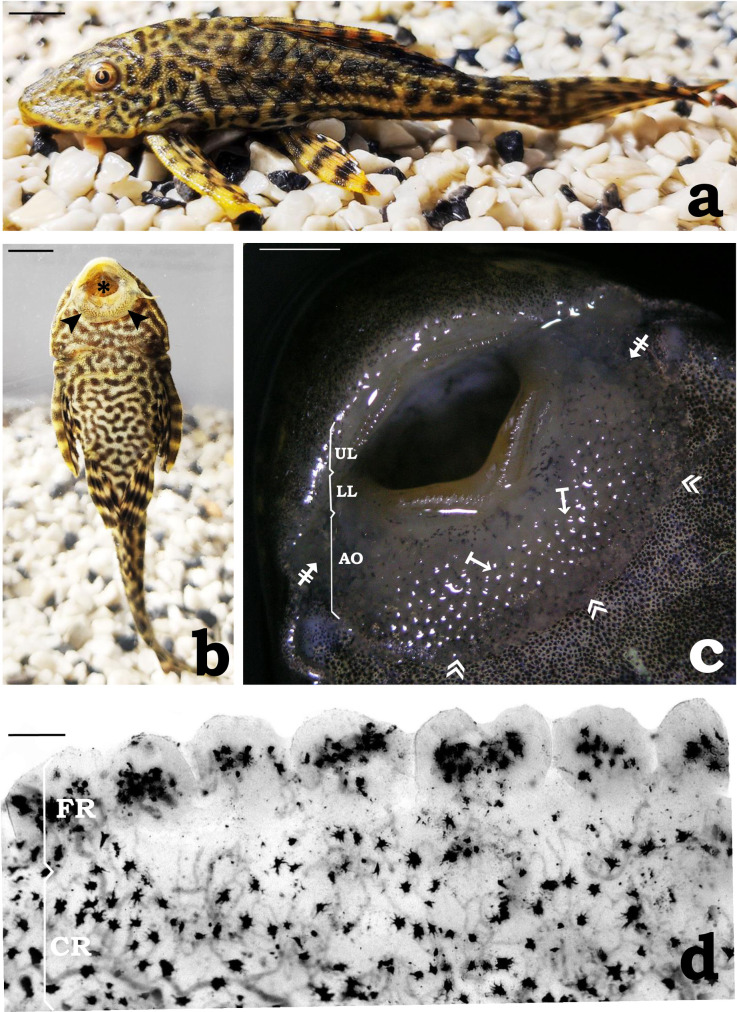
Photographs of *Pterygoplichthys disjunctivus.* (a) Lateral view of the fish (scale bar = 5 mm). (b) Ventral view of the fish showing the subterminal mouth opening (asterisk) and adhesive organ (AO) (arrowheads) (scale bar = 5 mm). (c) Similar to (Fig 1b), at higher magnification. Note that the AO is distinguished into the central region (arrows from the bar) and flap-like peripheral region on its posterior side (double arrowheads) and lateral sides (barred arrows) (scale bar = 2 mm). (d) Part of the unstained AO in the whole mount showing central and flap-like peripheral regions. Note the thin flap-like peripheral region is distinguished into lobulated structures. Further melanophores are visualized distributed irregularly in both the regions (scale bar = 20 µm). Abbreviations: UL, upper lip; LL, lower lip; FR, flap-like peripheral region; CR, central region.

Live specimens of *P. disjunctivus* [mean ± S. D. (standard deviation), standard length, Ls, 75 ± 5 mm; weight = 3.6 ± 2 g; n = 60] used in this study were obtained from a local fish supplier in Varanasi (Uttar Pradesh), India. Photographs [lateral view (Fig 1a) and ventral view (Fig 1b) of the whole fish; ventral view (Fig 1c) of the anterior part of the fish showing mouth opening, and AO] were captured in the laboratory using a digital camera (Nikon, Coolpix-E5400, Japan). Fish were acclimated to laboratory conditions in glass aquaria (80 × 50 × 40 cm) filled with water at a controlled room temperature (25 ± 2°C). The aquarium water was continuously aerated and filtered using an internal filter TED TABBIES Sobo, WP1000F (Signature Traders, China), and the fish were fed daily with a commercially available diet (Tokyu® pellets; Changwat Nakhon Pathom, Thailand) throughout the experiment. In addition, fish scrape their food from the aquarium wall. Water quality characteristics were determined as described by American Public Health Association (APHA), American Water Works Association (AWWA), and Water Pollution Control Federation (WPCF) [37]. The aquaria water quality parameters (mean ± S.D.) during the experiment were dissolved oxygen (6.79 ± 0.23 mg/L), pH (7.43 ± 0.04), alkalinity (228 ± 5.2 ppm), and hardness as CaCO_3_ (186.00 ± 3.46 mg/L).

### Histological organization

Fish *P. disjunctivus* used in this study acclimatized to laboratory conditions for at least 15 days prior to conducting the experiment were subjected to method for anaesthesia and/or analgesia. In this study, cold anaesthesia method reported by Mitttal & Whitear [38] was used to anaesthetise the fish. According to this method, the fish were kept in aquaria containing water at room temperature (25 ± 2°C). Crushed ice was then added to the water gradually and stirred at such a rate that the temperature of water fell by few degrees every few minutes (min.). At 10 ± 1°C, fish get anaesthetised. Fish under anaesthesia shows temporary loss of sensation, often including loss of consciousness, become immobile and unresponsive to touching or pricking. From the anaesthetised fish, tissue samples (approx. 6 × 8 mm) from the AO were excised, rinsed in physiological saline, and fixed in appropriate fixatives.

After sampling, the fish were subjected to euthanasia using a rapid cooling method following Valentim et al. [39]. In this method, the temperature of the water in which fish were kept was rapidly dropped to 2–4°C, by adding crushed ice. This method is considered a standard method in veterinary practice to provide a humane end to an animal’s life [40]. It induces a quicker and irreversible death and alleviates suffering by reducing or lessening the intensity of pain and distress [40].

The fixed tissue samples were dehydrated in a graded series of ethanol in ascending concentrations, cleared in xylene, and embedded in paraffin wax. Tissue sections were cut at 6 µm (approx.) using a Rotary Microtome (Model RM 2125RT; Leica Microsystems, Bensheim, Germany), mounted on glass slides, and dried in an oven at 37°C overnight. To examine histological organization, tissue samples were fixed in Bouin’s fluid. Deparaffinized sections were hydrated through a graded series of ethanol in descending concentrations to water and stained with Ehrlich’s hematoxylin and eosin (H/E) [41]. Tissue samples fixed in Carnoy’s fluid were deparaffinized and stained with periodic acid/Schiff (PAS) [42], alcian blue at pH 2.5, (AB2.5) [43], and AB2.5/PAS [44] to identify goblet cells.

Briefly, for PAS staining, the tissue sections were hydrated, oxidized in 0.5% aqueous periodic acid for 10 min, rinsed in distilled water, and immersed in Schiff’s reagent for 8 min. This was followed by three sequential rinses in freshly prepared sulphite solution (1% sodium metabisulphite in 0.1N HCl). Sections were then washed under running tap water for 15 min, dehydrated through graded alcohols, cleared in xylene, and mounted using distrene dibutylphthalate xylene (DPX).

For AB2.5 staining, the sections were rinsed in 3% acetic acid, stained with 1% alcian blue (pH 2.5) for 25–30 min, rinsed again in 3% acetic acid, and washed under running tap water for 20–25 min. Subsequent dehydration, clearing, and mounting were performed as described for PAS staining.

To determine the proteinaceous nature of secretory contents in serous goblet cells (SGCs), protein-bound NH₂ groups were identified using the Ninhydrin-Schiff method [45], whereas tyrosine residues were detected using Millon’s method [46]. Helly’s fluid-fixed sections were stained with Mallory’s Triple Stain (MTS) [47] to detect the acidophilic contents of the cellular components of the epithelium.

Stained sections were examined using an Olympus BX53 Upright Digital Microscope (Tokyo, Japan). Images were captured using an Olympus DP26 color camera attached to the microscope and recorded using an Intel Core i5 computer (Hp, Compaq, USA). This study was conducted in accordance with the guidelines of the Central Animal Ethical Committee of Banaras Hindu University (BHU) Varanasi, India (Ref. BHU/DoZ/IAEC/2021–22/020 dated 15/02/2022).

### Whole mount

Tissue samples (approx. 6 × 8 mm) from the AO were collected from anesthetised fish. The samples were rinsed in physiological saline and fixed in Carnoy’s fluid. Unwanted muscles from the underside of the AO were carefully removed using fine forceps and needles under a stereoscopic microscope (Nikon, Nippon Kogaku K. K., Tokyo, Japan). Tissues were divided vertically into two pieces. One piece was processed further as unstained tissue, and the other piece was stained with PAS [42], dehydrated in a graded ethanol series, and cleared in xylene. Whole-mount preparations were prepared using DPX and examined under a Leica DM750 Microscope (Leica Microsystems, Germany). Images were captured with Flexacam C3 color camera attached to the microscope and recorded on an Intel Core i7 computer (HP, Compaq, USA).

### Scanning electron microscopy

Samples (approx. 5 × 5 mm) from the AO of anesthetised fish were excised, rinsed in physiological saline (pH 7.4), and then briefly dipped 4–5 times in a 0.1% S-carboxymethyl-L-cysteine solution to remove surface mucus, following Whitear & Moate [48]. All subsequent steps, including fixation, post-fixation washing, and dehydration, were performed in cold (at 4°C). The tissues were then fixed in 3% glutaraldehyde prepared in 0.1 M sodium cacodylate buffer (pH 7.4) for 4 hours (h). After fixation, the tissues were rinsed in dilute buffer solution (1:1 mixture of 0.2 M sodium cacodylate buffer and distilled water). The tissues were dehydrated in an ethanol-acetone mixture (3:1, 1:1, and 1:3) in ascending ethanol, followed by anhydrous acetone and critical point dried using a Critical Point Dryer (CPD) (E3100 Series, Quorum Technologies Ltd., Polaron) using liquid carbon dioxide as the transitional fluid. Tissues were glued to stubs using conductive silver preparation (Eltecks Corporation, India), coated with gold using a Sputter Coater (BU015331-T, Baltec, Switzerland), and examined under SEM (EVO 18, Zeiss). at the Sophisticated Analytical Instrumentation Facility (SAIF), Department of Anatomy, All India Institute of Medical Sciences (AIIMS), New Delhi, India. Data were recorded and analyzed using an Intel Pentium IV D computer (Model dx2280 MT, HP Compaq).

### Proteomic analysis

Tissue samples (approx. 100 mg) were excised from the AO. Simultaneously, for comparison, tissue samples (approx. 100 mg each) from two regions of the body skin were also excised. These were the skin from (a) the ventral region of the body, located immediately posterior to the opercular opening between the bases of the pectoral fins, and (b) the dorsal region situated near the dorsal fin behind the head. Furthermore, the processing of each of these samples was performed separately. Each tissue sample was homogenized in 1.5 ml of buffer containing 8M urea, 0.1M Tris-HCl (pH 8.5), 2% (w/v) dithiothreitol, and protease inhibitors using a homogenizer (IKA, India). Homogenates were centrifuged at 10,000 revolutions per minute (RPM) for 15 min at 4°C. The supernatant was decanted and chilled acetone in a 1:3 ratio was added. The mixture was then kept at −20°C for 20 h to allow protein precipitation. After 20 h, the mixture was centrifuged at 1000 RPM for 1 min. Consequently, the protein pellets settled at the bottom of the centrifuge tube. The supernatant was discarded, and the resultant protein pellets obtained through acetone precipitation were resuspended in 160 μl of rehydration buffer containing 7M urea, 2M thiourea, and 20mM dithiothreitol. Aliquots of rehydration buffer containing dissolved protein pellets were divided into two parts. One part of these aliquots was used to determine the protein concentration in each sample and to perform Sodium Dodecyl Sulfate-Polyacrylamide Gel Electrophoresis (SDS-PAGE) analysis. The second part of the aliquots was used for quantification and identification of proteins in each sample using a HRAMS at the Sophisticated Analytical and Technical Help Institute (SATHI) of BHU, Varanasi, India.

#### Protein estimation and SDS-PAGE.

The protein concentration in each tissue sample (AO, ventral, and dorsal) was determined by Bradford protein assay using bovine serum albumin (BSA) as a standard, following the cold spring harbor protocol (http://cshprotocols.cshlp.org). SDS-PAGE was performed using a mini electrophoresis system (Genie, Bangalore, India) on 15% polyacrylamide gels. Each well was loaded with 20 µg of protein and electrophoresis was continued until the dye front reached the bottom. The gels were then stained with 0.05% Coomassie Brilliant Blue R-250. The stained gels were treated with a mixture of 40% methanol and 10% acetic acid to differentiate and visualize different protein bands. Molecular weight markers (CatP7719S; New England Biolabs, USA) were used as standards for the estimation. Images of the gel were captured using a digital camera system (Nikon Coolpix-E5400, Japan) and recorded using an Intel Core i7 computer (Xiaomi, China).

#### Protein quantification and identification using HRAMS.

**In-solution tryptic digestion and mass spectrometry data acquisition:** One milligram of protein was homogenized in 100 μl of 7M urea buffer (7M urea in 50mM ammonium bicarbonate). Reduction was performed with 50 μl of 200 mM dithiothreitol for 1 h at 25°C, followed by alkylation with 50 μl of 200 mM iodoacetamide at room temperature for 30 min in the dark. Subsequently, 100 μl of 1 mM calcium chloride was added and mixed. The solution was then incubated at room temperature for 10 min. to adjust the pH above 7.0, and the urea concentration to ~0.5 M. Sequencing grade modified trypsin (Promega Corporation, USA) was added at a 1:50 ratio (trypsin: protein), mixed, and incubated at 37°C for 16–20 h. The pH was lowered using 0.1% formic acid and desalted using a C18 spin column (Pierce™ Peptide Desalting Spin Columns) following the manufacturer’s protocol (Thermo Fisher Scientific, Waltham, Massachusetts, USA).

Protein quantification was performed by Label-Free Quantification using Proteome Discoverer 3.1.0.638 (Thermo Scientific). For comparison, AO was used as samples and skin from the ventral and dorsal regions of the body were used as controls. Samples and controls were separated using an UltiMate^TM^ 3000 RSLCnano Series for 120 min. Peptides (1 µg) were loaded onto a C18 trap column (Acclaim PepMap100) and separated on a C18 analytical column (PepMapTM RSLC C18) using a gradient buffer (85% acetonitrile + 0.1% Formic Acid). The peptides were analysed with an Orbitrap Eclipse Tribrid Mass Spectrometer (Thermo Fisher Scientific, USA) using the Orbitrap-Ion Trap hybrid method. In the Orbitrap-Ion Trap hybrid method, both Orbitrap and ion trap analyzers are used for complementary analysis. Mass spectrometry data were acquired from the top 20 scans by selecting precursor ions (300–1650 m/z) for Higher Energy Collision Dissociation fragmentation. Data-dependent MS2 was performed using the quadrupole isolation mode with a 1.5 m/z window and 30% collision energy, detected by an ion trap detector.

**Mass spectrometer data analysis:** The total ion chromatograms of all three samples were consistent. Data analysis was performed using Proteome Discoverer 3.1.0.638 (Thermo Scientific), and identification was restricted to the taxonomic group Actinopterygii with a signal-to-noise ratio of 1.5 or above. Target FDR (strict) < 0.01 was considered to ensure reliable quantitative results and proteins with significant hits (p-adjusted value ≤ 0.05) with a score above the threshold level, and at least one unique peptide sequence was identified.

**Gene ontology and protein-protein interaction:** Gene Ontology (GO) terms for the identified proteins were obtained from the UniProtKB (www.uniprot.org) and EMBL-EBI (www.ebi.ac.uk) databases using Proteome Discoverer 3.1.0.638 (Thermo Scientific). GO enrichment analysis was conducted using the Enrichr software (http://maayanlab.cloud/Enrichr/), following the method described by Chen et al. [49] and Hsiao et al. [50], to categorize proteins based on biological processes, cellular components, and molecular functions. Due to the lack of species-specific data for *P. disjunctivus*, human orthologs of the proteins were used for enrichment analysis.

A protein-protein interaction map was generated using STRING v12.0, (www.string-db.org) following Szklarczyk et al. [51], with medium confidence limits (<0.400) and an average node degree of 4.1. Human orthologs were used as inputs, as most of the proteins were not well annotated in teleost species. STRING assigns protein names from databases, such as Biocarta, BioCyc, GO, KEGG, and Reactome. The image displays networks of proteins, where individual proteins are represented by nodes and their connections are indicated by edges. Nodes are depicted as either filled or empty, with filled nodes representing proteins with known structures and empty nodes representing those without known structures. The edges illustrate potential functional associations regardless of direct physical binding. The strength of the interaction between proteins was indicated by the number of edge lines linking the nodes.

## Results

**Fig 2:**
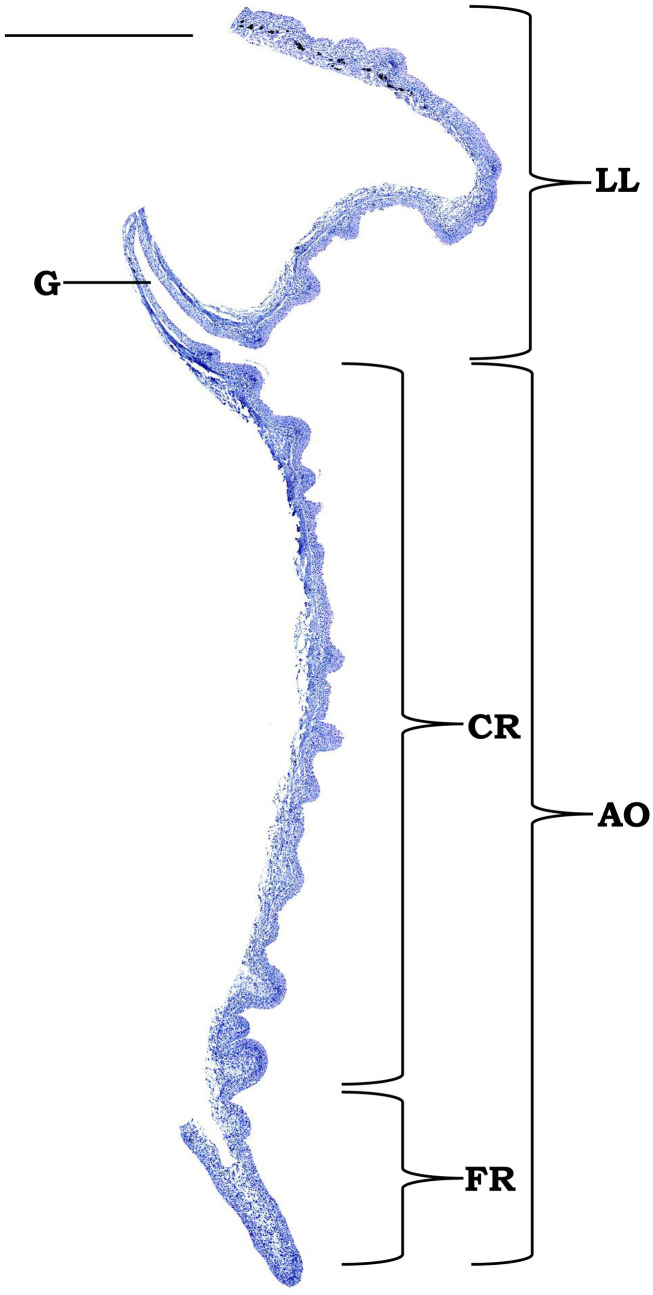
Photomontage of cross sections of the lower lip and associated structures of *Pterygoplichthys disjunctivus* (haematoxylin and eosin [H/E]) (scale bar = 600 µm). Abbreviations: LL, lower lip; G, groove; CR, central region; FR, flap-like peripheral region; AO, adhesive organ.

In *P. disjunctivus* (Fig 1a)*,* the mouth is sub-terminal (Fig 1b). It is bordered dorsally by the upper lip, which covers the upper jaw, and ventrally by the lower lip, which covers the lower jaw (Fig 1c). Adjacent to the ventral side of the lower lip, a prominent specialized structure, the AO was observed. AO is characteristically plate-like, large, and, to some extent, semicircular in its outline (Fig 1c). It can be divided into a major, conspicuous, well-developed central region and a relatively narrow, flap-like peripheral region on its posterior and lateral sides. The margins of flap-like peripheral region appear lobulated (Fig 1d). Adjacent lobules are separated by deep indentation. In between the AO and the lower lip, a deep groove is observed (Fig 2).

### Histological organization of the adhesive organ

**Fig 3:**
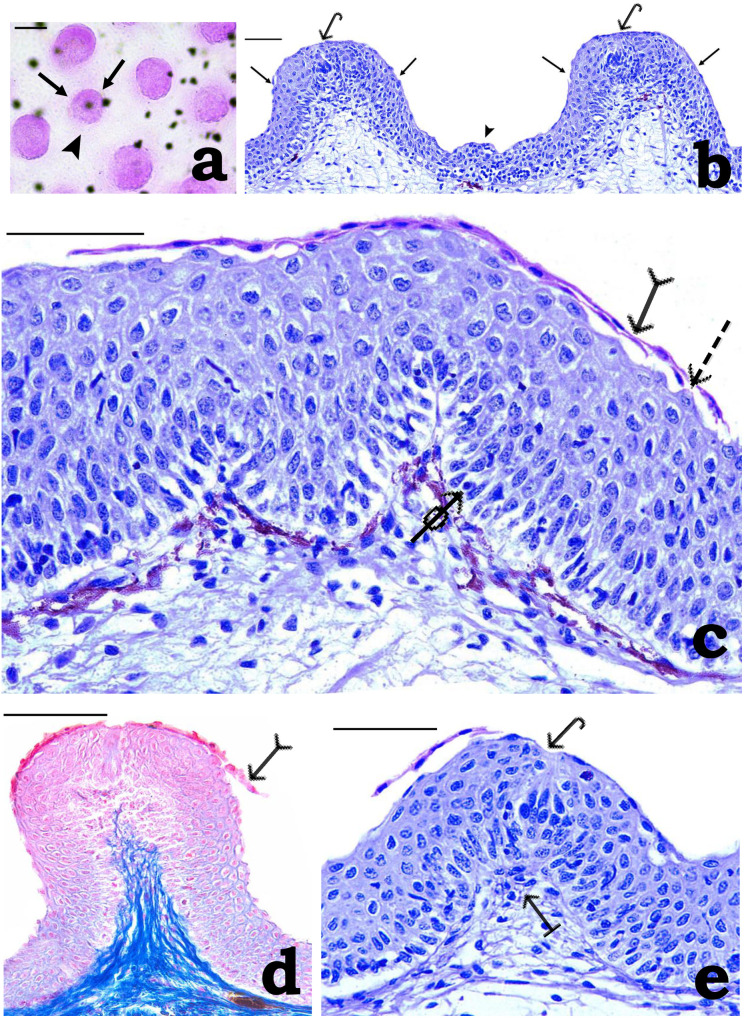
Photomicrographs of (a) whole mount and (b-e) cross-sections of adhesive organ (AO) of *Pterygoplichthys disjunctivus.* (a) Part of AO stained with periodic acid/Schiff showing mound-like tubercles (arrows) separated by deep furrows (arrowhead). Note melanophores distributed irregularly are visualized (scale bar = 20 µm). (b) The epithelium shows characteristic mound-like tubercles (arrows) separated by deep furrows (arrowhead). Note a pear-shaped taste bud (arrows with hook) located at the summit of each tubercle (scale bar = 20 µm). (c) Part of the mound-like tubercle epithelium at higher magnification. Epithelial cells appear flattened and often modified into prominent spine-like unculi (dashed arrow) in the outer layer; these in the superficial layer are often visualized detached and slough off (arrow with tail) from the underlying epithelial cells. In between the basal layer epithelial cells, darkly stained lymphocytes (arrow with circle) could be located (scale bar = 100 µm). (d) Epithelial cells in the OL of the mound-like tubercle epithelium appear flattened and slough off (arrow with tail) from the underlying epithelial cells, which stain bright red with Mallory’s triple stain (scale bar = 40 µm). (e) A pear-shaped taste bud (arrow with hook) located at the summit of the mound-like tubercle and supported by the papilla (arrow from the bar) from the underlying tissues (scale bar = 40 µm).

The free surface of the AO shows characteristic irregularly distributed mound-like tubercles separated by deep furrows (Fig 3a and 3b). The epithelium covering both tubercles and furrows is stratified and consists of epithelial cells arranged in several tiers. The epithelium in both of these regions can arbitrarily be divided into the outer layer (OL), the middle layer (ML), and the basal layer (BL).

In mound-like tubercles, the epithelium is represented by epithelial cells arranged in 2–4 tiers in the OL, 6–8 tiers in the ML, and a single layer in the BL (Fig 3c). The epithelial cells in the superficial layer (outermost layer of the OL) are greatly flattened with compact and flattened nuclei and are often detached and slough off in the form of a sheet from the underlying 2^nd^ tier epithelial cells of the OL (Fig 3c). With H/E, the cytoplasmic contents of these cells stained pink, while the nuclei stain deep blue (Fig 3c). Further, with MTS, these cells color bright red (Fig 3d). The 2^nd^ tier epithelial cells appear rectangular or vertically flattened. These cells often appear modified into prominent spine-like unculi, usually conical with a broad base that gradually tapers towards the apical end (Fig 3c). In the OL and the ML, the epithelial cells in general, appear irregular polygonal. In the BL, the cells are columnar. In H/E, epithelial cells throughout the epithelium generally appear compactly arranged, each with a central healthy-appearing rounded nucleus with distinct chromatin material and nucleoli. Between the BL epithelial cells, rounded spaces – the lymphatic spaces each containing rounded lymphocytes, which stained dark blue in H/E, could be located (Fig 3c). At the summit of each mound-like tubercle, a typical pear-shaped taste bud (TB) could be located (Fig 3b and 3e). Each TB is supported by a connective tissue papilla arising from tissues underlying the epithelium (Fig 3e), through which TBs receive nerve and blood supply.

**Fig 4:**
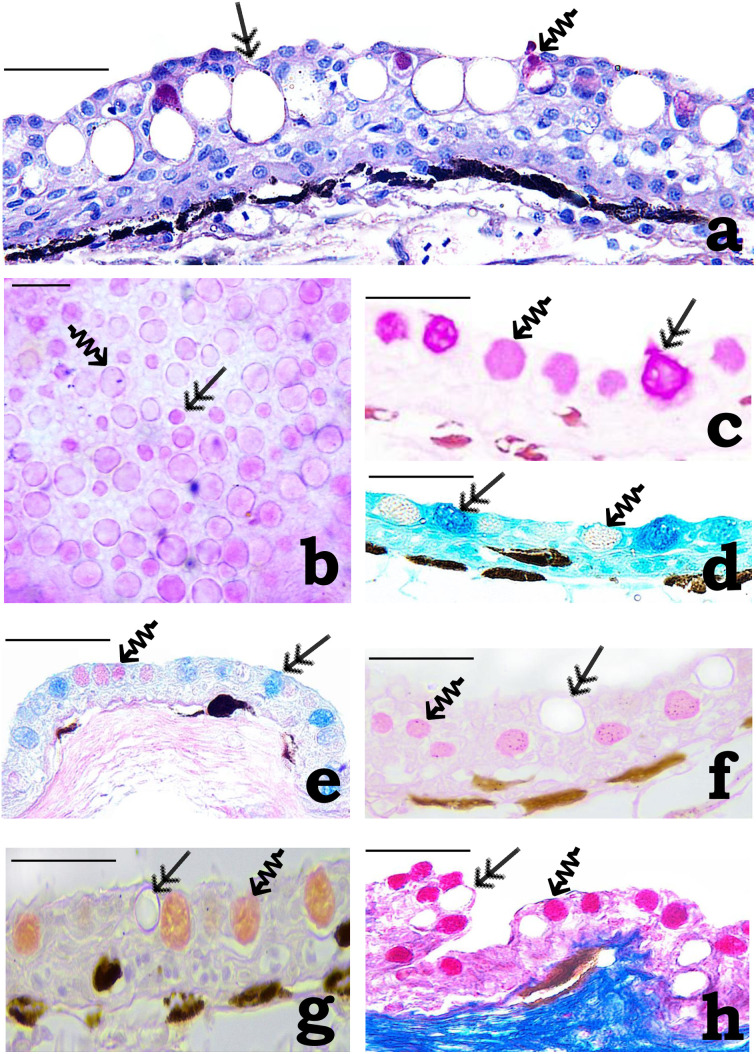
Photomicrographs of (a, c-h) cross-sections and (b) whole mount of adhesive organ (AO) of *Pterygoplichthys disjunctivus.* (a) Interspersed between the epithelial cells of the furrow epithelium, the mucous goblet cells (MGCs) (double-headed arrow) and the serous goblet cells (SGCs) (squiggle arrow) are observed (scale bar = 40 µm). (b) A part of AO stained with periodic acid/Schiff (PAS) showing irregular distribution of the MGCs (double-headed arrow) and the SGCs (squiggle arrow) (scale bar = 20 µm). (c) MGCs (double-headed arrow) stain magenta, and SGCs (squiggle arrow) stain pink with PAS (scale bar = 40 µm). (d) MGCs (double-headed arrow) stain turquoise and the SGCs (squiggle arrow) remain unstained with alcian blue at pH 2.5 (AB2.5) (scale bar = 40 µm). (e) MGCs (double-headed arrows) stain blue with purple tinge and the SGCs (squiggle arrow) stain pink with AB2.5/PAS (scale bar = 40 µm). (f) MGCs (double-headed arrows) remain unstained and SGCs (squiggle arrow) stain pink with Ninhydrin-Schiff (scale bar = 40 µm). (g) MGCs (double-headed arrows) remain unstained and SGCs (squiggle arrow) stain orange with a red tinge with Millon’s method (scale bar = 40 µm). (h) MGCs (double-headed arrows) remained unstained, and SGCs (squiggle arrow) stain bright red with Mallory’s triple stain (scale bar = 40 µm).

In contrast, in the furrow epithelium, the epithelial cells are arranged in 1–2 tiers in the OL, 2–3 tiers in the ML, and a single row in the BL. Epithelial cells in the OL often appear flattened or wedge-shaped; rounded, or irregular polygonal in the ML and low-columnar or cuboidal in the BL (Fig 4a). Lymphatic spaces containing lymphocytes could be located between BL epithelial cells at long intervals. Further, in the furrow epithelium, goblet cells could be observed distributed at irregular intervals (Fig 4a and 4b). The goblet cells are of 2 types: the mucous goblet cells (MGCs) and the SGCs (Fig 4a and 4b). The nuclei of these cells are basal, flattened, crescentic, or rounded. The secretory contents of MGCs either remain unstained or appear feeble blue with H/E (Fig 4a). These cells stain magenta with PAS (Fig 4b and 4c), turquoise with AB2.5 (Fig 4d), and blue with a purple tinge with AB2.5/PAS (Fig 4e), indicating the presence of glycoproteins in their secretory contents. The secretory contents of SGCs, in contrast, are finely granular and strongly eosinophilic with H/E (Fig 4a). These cells stain pink with Ninhydrin-Schiff (Fig 4f), orange with red tinge with Millon’s method (Fig 4g), and bright red with MTS (Fig 4h), indicating the proteinaceous nature of the contents of these cells. The pink stain with PAS (Fig 4b and 4c) and AB2.5/PAS (Fig 4e), further indicates the presence of small amount of glycoproteins as well in their contents.

### SEM of the adhesive organ

**Fig 5:**
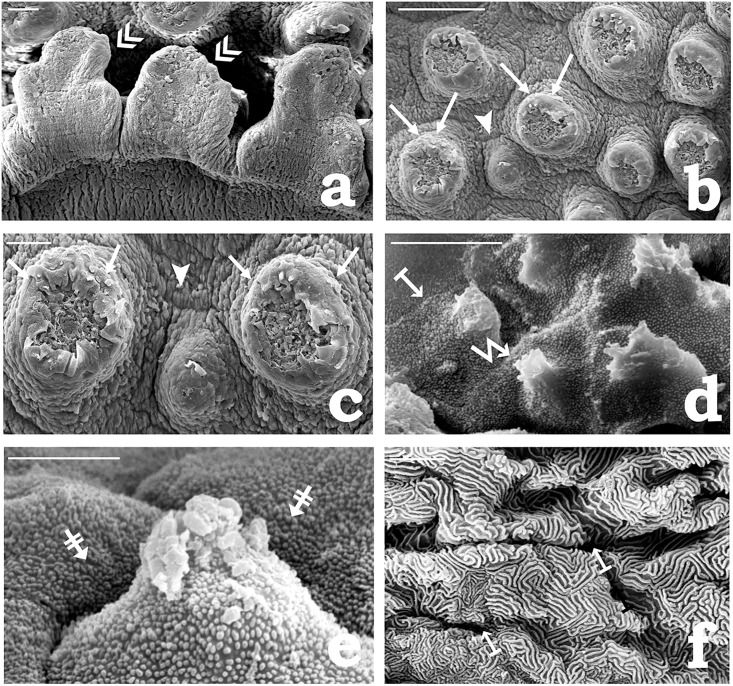
Scanning electron micrographs showing surface ornamentation of the adhesive organ of *Pterygoplichthys disjunctivus.* (a) Flap-like peripheral region (double arrowheads) of the adhesive organ (scale bar = 20 µm). (b) The central region of the adhesive organ consists of irregularly distributed mound-like tubercles (arrows) separated by deep furrows (arrowhead) (scale bar = 100 µm). (c) Similar as (Fig 5b), at higher magnification (scale bar = 20 µm). (d) Free surface of epithelial cells (arrow from the bar) of the mound-like tubercle, modified into a spine-like structure, the unculus (zigzag arrow) (scale bar = 2 µm). (e) Higher magnification of each epithelial cell of the mound-like tubercle showing punctate microridges (barred arrows), which gives a grainy appearance (scale bar = 2 µm). (f) Furrow region showing the free surfaces of epithelial cells with elongated and fragmented microridges. Small, rounded openings of goblet cells (arrows from the bar) are observed at irregular intervals between 2-3 epithelial cells (scale bar = 2 µm).

In *P. disjunctivus*, the thin flap-like peripheral region of the AO on its posterior and lateral sides is distinguished into lobulated structures separated by deep indentation (Fig 5a). It often gets rolled up during the tissue preparation for SEM. The central region of AO consists of an irregularly distributed mound-like tubercle separated by deep furrows (Fig 5b and 5c). Each mound-like tubercle has a wide proximal base, and appears relatively narrow at the apical end. It is covered with a mosaic pavement of irregular polygonal epithelial cells with distinct and prominent boundaries (Fig 5d). The free surface of each epithelial cell shows punctate microridges, giving a grainy appearance to the surface (Fig 5e). At intermittent intervals, epithelial cells could be located with their free surface modified into a characteristic spine-like structure, the unculus (Fig 5d). Further, each mound-like tubercle at its apical end characteristically shows a rounded opening, the taste pore, through which a TB projected onto the surface could be observed (Fig 5b and 5c). The surface epithelial cells around the taste pore often appear partially detached and appeared to be exterminated from the underlying tissues (Fig 5b and 5c). In the furrow region, the epithelium appears differentiated into slightly raised, extensive, longitudinally ridged, and separated by shallow grooves (Fig 5f). Longitudinal ridges frequently exhibit shallow depressions at irregular intervals. The epithelial surface, similar to that of the mound-like tubercle, is covered with a mosaic pavement of irregular polygonal cells. In contrast, the free surfaces of these cells exhibited elongated and fragmented microridges, forming irregular maze-like patterns. In between 2–3 epithelial cells, small, rounded apertures were observed at irregular intervals (Fig 5f). These apertures could be the openings of the MGCs or the SGCs at the surface.

### Qualitative analysis: SDS-PAGE

**Fig 6:**
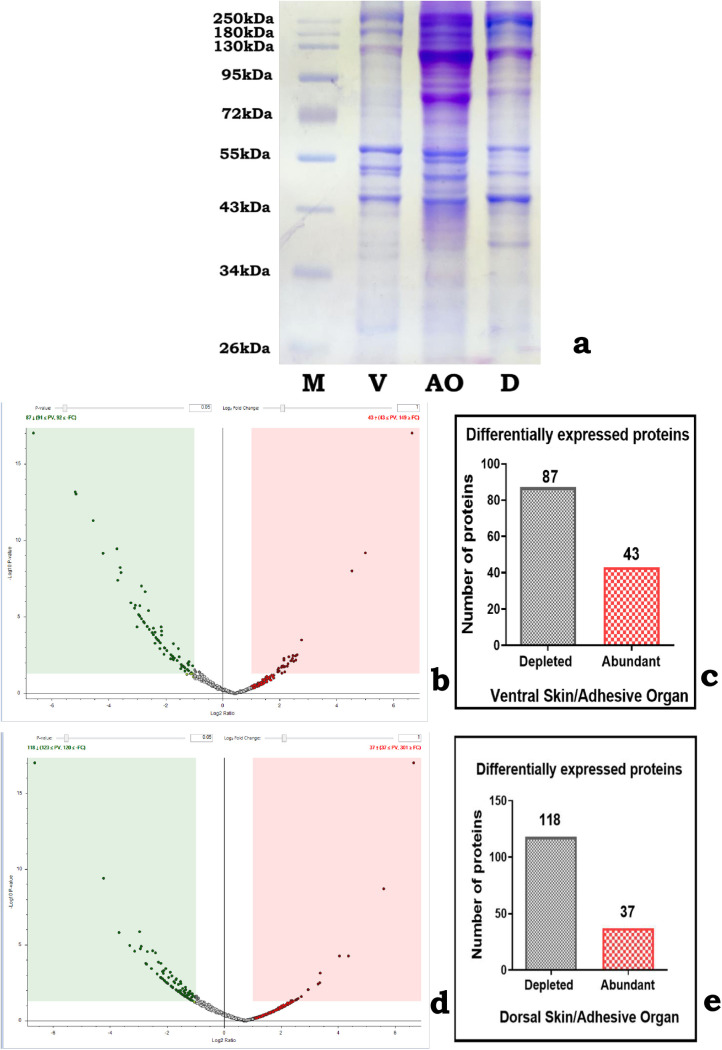
(a) SDS-PAGE profiles of the protein extract from the adhesive organ and ventral and dorsal skin regions of *Pterygoplichthys disjunctivus.* (b) Volcano plot illustrating the distribution of differentially expressed proteins between the ventral skin (control) and adhesive organ (AO) (sample). The x-axis indicates the log2 fold change, which represents the magnitude of differential expression, whereas the y-axis reflects the statistical significance of these changes through the -log10 p-value. Proteins with significant differential expression are marked by red dots (abundant in AO) and green dots (depleted in AO). (c) Bar graph summarizing the number of differentially expressed proteins of Fig 6b. A total of 87 proteins are significantly depleted in AO compared to ventral skin, whereas 43 proteins are significantly abundant in AO. (d) Volcano plot illustrating the distribution of differentially expressed proteins between the dorsal skin (control) and adhesive organ (AO) (sample). The x-axis indicates the log2 fold change, which represents the magnitude of differential expression, whereas the y-axis reflects the statistical significance of these changes through the -log10 p-value. Proteins with significant differential expression are marked by red dots (abundant in AO) and green dots (depleted in AO). (e) Bar graph summarizing the number of differentially expressed proteins of Fig 6d. A total of 118 proteins are significantly depleted in the AO compared to dorsal skin, whereas 37 proteins are significantly abundant in the AO. Abbreviations: V, ventral skin; AO, adhesive organ; D, dorsal skin; M, molecular weight marker.

SDS-PAGE analysis of the protein extracts from AO and skin of the ventral and dorsal regions of *P. disjunctivus* reveals distinct protein profiles (Fig 6a). AO exhibited higher protein expression than the ventral and dorsal skin. Notably, AO displayed well-defined prominent bands in the range 72–250 kDa. These are less prominent or absent in other skin regions. Furthermore, the AO protein bands at 80 kDa and 130 kDa are significantly more intense and prominent than those in the ventral and dorsal regions. Protein bands at 44 kDa, 56 kDa, 180 kDa, and 250 kDa are observed in all three regions. Additionally, certain unique protein bands are identified: 52 kDa and 55 kDa in the AO, 54 kDa in the ventral skin, and 38 kDa in the dorsal skin. In general, protein bands below 36 kDa are less resolved in all the regions.

### Quantitative analysis of differentially expressed proteins (DEPs)

#### Ventral skin and adhesive organ.

Quantitative proteomic analysis revealed significant differences in protein expression profiles between ventral skin (control) and AO (sample). AO exhibited a distinct set of abundant (red dots) and depleted (green dots) proteins compared with the ventral skin, as shown in the volcano plot (Fig 6b). Among the 130 DEPs identified, 43 are significantly abundant in the AO, whereas 87 are notably depleted compared with those in the ventral skin (Fig 6b and 6c). The details of the abundant proteins are provided in S1 Table and those of the depleted proteins are listed in S2 Table.

#### Dorsal skin and adhesive organ.

Distinct differences are observed in the protein expression between the dorsal skin (control) and AO (sample). The volcano plot highlights both abundant (red dots) and depleted (green dots) proteins in the AO compared with those in the dorsal skin (Fig 6d). Of the 155 proteins showing differential expression, 37 are markedly abundant in the AO and 118 are significantly depleted relative to those in the dorsal skin (Fig 6d and 6e). Details of the abundant proteins are provided in S3 Table and those of the depleted proteins are listed in S4 Table.

#### Categorization of the abundant proteins of the adhesive organ.

The 80 abundant proteins in the AO group (43 relative to the ventral skin and 37 relative to the dorsal skin) are organized in a descending order of significance based on adjusted p-values ≤ 0.05. Proteins related to adhesion – Periplakin (PPL), Desmoplakin (DSP), and Filamin-A-like (FLNA); and related to keratinization – Keratin, type II cytoskeletal 8-like (KRT8) and Keratin, type I cytoskeletal 19-like (KRT19) shows highly significant adjusted p-values. The full list of abundant proteins in AO, ranked in descending order of their significance, is provided in S5 Table. S6 Table summarizes these proteins and their functions retrieved manually from UniProtKB and EMBL-EBI databases.

#### Gene ontology and interaction networks of the abundant proteins in the adhesive organ.

**Fig 7:**
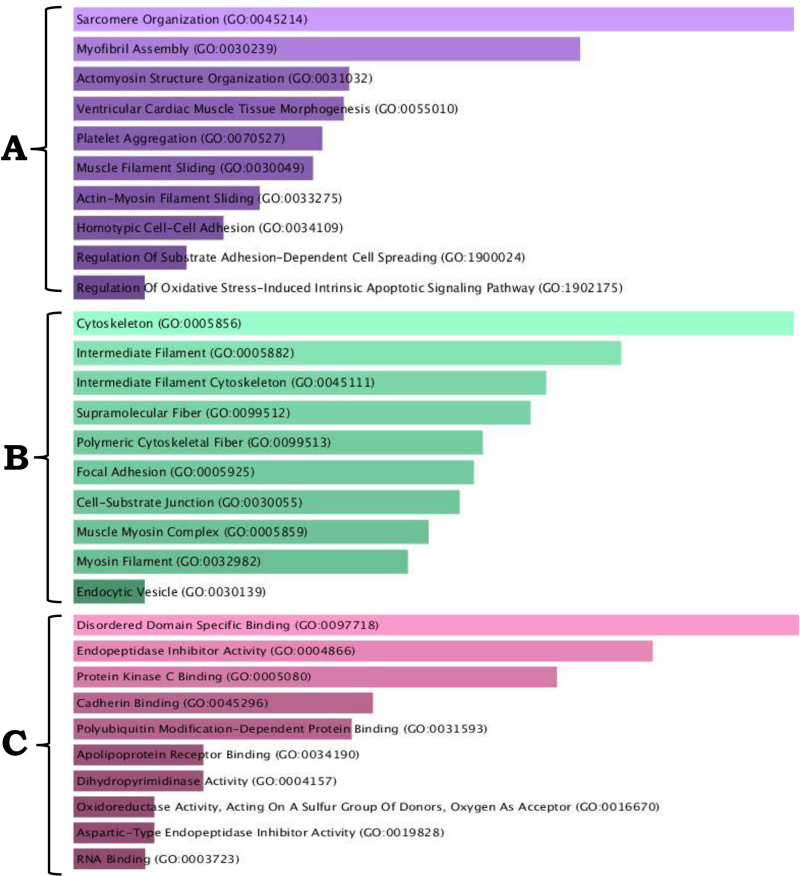
Gene Ontology enrichment analysis of 80 abundant proteins identified in the adhesive organ of the *Pterygoplichthys disjunctivus.* Proteins are categorized as (A) biological processes, (B) cellular components, and (C) molecular functions.

GO enrichment analysis, conducted using Enrichr software, classified the 80 abundant proteins identified in AO into three primary categories: Biological Processes, Cellular Components, and Molecular Functions (Fig 7). The most significantly enriched biological processes are sarcomere organization, myofibril assembly, and actinomyosin structural organization. The cellular component category includes the cytoskeleton, intermediate filaments, supramolecular fibres, and focal adhesion complexes. The main molecular functions include disordered domain-specific binding, endopeptidase inhibitor activity, and protein kinase C and cadherin binding.

**Fig 8:**
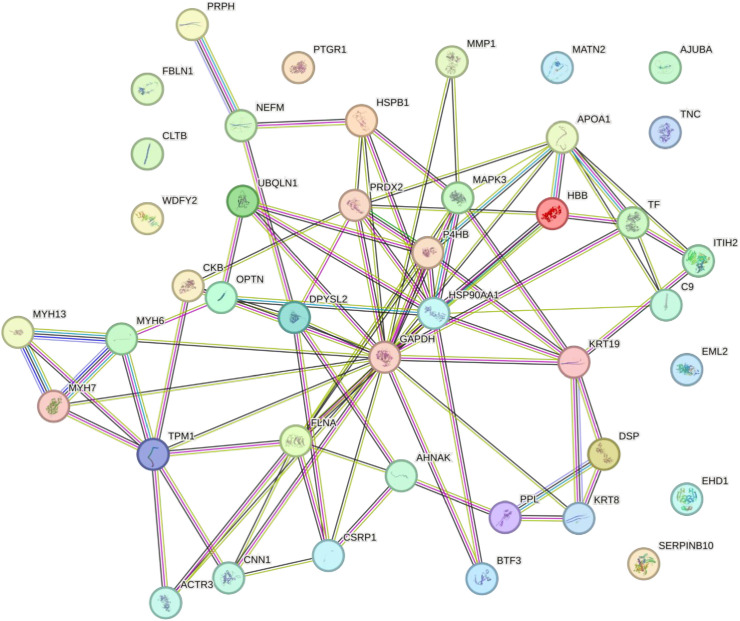
Protein-protein interaction map generated using STRING v.12.0, with medium confidence limits (<0.400) and human orthologs as inputs. Proteins are represented by nodes and their connections are indicated by edges. Nodes are depicted as either filled or empty, with filled nodes representing proteins with known structures and empty nodes representing those without known structures. The edges illustrate potential functional associations, regardless of direct physical binding. The strength of the interaction between proteins was indicated by the number of edge lines linking the nodes.

A protein-protein interaction map is constructed using STRING v12.0 (Fig 8). The resulting interaction network comprised 42 nodes and 86 edges. The network highlighted the interactions between adhesive proteins (DSP, PPL, AHNAK, FLNA, and CNN) and keratinized proteins (KRT8 and KRT19). The network also included the myosin heavy chain protein family (MYH6, MYH7, MYH13), cytoskeletal proteins (ACTR3, GAPDH, DPYSL2, and TPM1), enzymatic proteins (PRDX2, MMP1, P4HB), transport proteins (APOA1, C9, TF), and proteins associated with metabolic and binding processes (CKB, UBQLN1, BTF3). Other proteins that are not interconnected in the interaction network include SERPINB10, CLTB, PTGR1, FBLN1, MATN2, AJUBA, TNC, EML2, EHD1, and WDFY2. Comprehensive details of the protein names and abbreviations used in the interaction network are provided in S7 Table. All the protein abbreviations are derived directly from STRING to ensure consistency with the database.

## Discussion

Fish exhibit a remarkable diversity of adhesion strategies to adopt for their challenging habitats and ecological environments. The present study on *P. disjunctivus,* a hillstream fish reports a unique structure, the AO on the ventral side of the head close to the lower lip. Presence of the AO is significant and it could be associated as an adaptation for adhesion of the fish. This may ensure a firm grip of the fish on the substrata, preventing it from being washed away under fast-flowing turbulent water currents in hill streams. Adhesive organs have also been reported in several other hill stream fish species. These include *Gyrinocheilus* sp. [52]; *Garra* sp. [23–25,53–56]; *Schizothorax richardsonii* [57]; *Glyptothorax* sp. [21,22,58,59]; *Pseudocheneis sulcatus* [60]; and *Gobiesox maeandricus* [20].

In *P. disjunctivus,* AO, like a suction disc could function to create a vacuum. At first, the mound-like tubercles on the surface of the AO could be pressed firmly against the substratum, involving contraction of the muscles underlying it. Subsequent relaxation of these muscles results in the creation of the vacuum. This could lead to AO adherence to the substratum. Further, longitudinal ridges separated by shallow grooves in the epithelium of the furrow regions, between the mound-like tubercles, could be associated with an increased surface area during the generation of a vacuum when pressed against the substratum. Additionally, the flap-like structure at the posterior and lateral margins of the AO could strengthen adhesion by creating a sealing mechanism that ensures firm grip on the substrate. The vacuum thus created could provide anchorage of the fish to the substratum effectively. This structural adaptation not only strengthens attachment by creating a strong seal but also serves as a protective barrier, preventing pathogen adherence and penetration into the underlying tissues. The adhesion strategy that relies on suction-based attachment reported in this study corroborates the findings of Das & Nag [56] and Ditsche et al. [20], who surmised the role of AO in suction attachment in *Garra gotyla gotyla* and *Gobiesox maeandricus*. Nevertheless, Geerinckx et al. [61], who did not describe the surface architecture and histological organization of the organ for attachment, also reported suction attachment to the substratum of *P. disjunctivus*. The emphasis in their study was on kinetic analysis to acquire insights into how respiration and attachment are combined.

In *P. disjunctivus* AO*,* presence of the spine like unculi, on the surface of the mound-like tubercles could be considered to reinforce firm anchorage of the fish to the substratum. These unculi increase the resistance to slippage, thereby enhancing the friction to maintain attachment. This view is consistent with that of Beckert et al. [62], who reported similar observations for *Echeneis naucrates*. Similarly, several researchers [63–65] in hillstream loaches, described that unculi interlock with vegetative or irregular surfaces on the substrate, thus facilitating adhesion. In addition to their mechanical function in assisting the fish to cling on the substratum, the unculi on the free surface of the AO, could serve to scrape food particles, such as diatoms, periphytons, algae, and vegetation from the substratum.

In *P. disjunctivus*, presence of the MGCs involved in the secretion of glycoproteins and SGCs, involved in the secretion of proteins, is noteworthy. A combination of the secretions of MGCs and SGCs on the surface of AO could be considered a biological adhesive and offer protective lubricating coatings. Lengerer & Ladurner [66] stated that adhesion mechanisms often rely on specialized proteins and polysaccharides,displaying adhesive solutions that have evolved to meet the demands of aquatic life. Glycoproteins and proteins have also been reported in adhesive secretions of many echinoderms [67], worm-like invertebrates [68], and snails, *Helix aspersa* [69] and *Achatina fulica* [70].

Proteomic analysis of *P. disjunctivus* showed that AO had significantly higher levels of proteins associated with adhesion. These include PPL, DSP, and FLNA proteins.

Elevated levels of PPL, an intermediate filament associated protein in AO of *P. disjunctivus,* is significant. Guzmán et al. [71] explore the function of PPL and stated that it connects cytoskeletal elements to membrane-bound proteins and provides cellular strength under mechanical stress. In view of this, higher levels of PPL in the AO of *P. disjunctivus* could be considered to play a crucial role in tissue robustness and stability, which is essential for surviving the high-flow water of hill streams. This adaptation in *P. disjunctivus* may have evolved in response to ecological pressure, which requires strong adhesive strength and resilience for substrate attachment. Further, Guzmán [71] and Sapra & Medalia [72] highlighted that PPL enhances physical integrity and adaptability, emphasizing its evolutionary significance across vertebrates from fish to mammals.

In *P. disjunctivus* AO, conserved and upregulated levels of DSP, a vital desmosome component, could be associated with enhanced cell-cell adhesion, providing a selective advantage by strengthening the tissue against detachment and mechanical stress. DSP enhances cellular adhesion by linking intermediate filaments to intercellular adhesion sites, thus creating a supracellular scaffold that distributes mechanical force throughout the tissue [73,74]. The evolutionary significance of DSP across vertebrates, such as *Cyprinus carpio* [75] and *Homo sapiens* [76], aligns with and supports the findings of the present study regarding its critical role in enhancing tissue adhesion and resilience within adhesive organ. This adaptation illustrates how evolutionary pressure shaped vertebrate proteins to support both adhesion and resilience [77].

FLNA, abundantly expressed in the AO of *P. disjunctivus*, may enhance extracellular matrix elasticity, resilience, and cell adhesion [78]. Nakamura et al. [79] suggested its role in adapting to mechanically challenging habitats.

Presence of KRT8 in significant concentrations in the AO of *P. disjunctivus*, is interesting. The role of the KRT8 protein in tissue stability and adaptability has been well documented by several workers [80–84], making it essential for survival in diverse environments. The presence of the KRT8 protein, together with proteins associated with adhesion in AO, could be considered an evolutionary advantage for supporting strong adhesion and mechanical stability in rigorous habitats. This corroborates the report in *Danio rerio* [85], where KRT8 has been reported to reinforce cellular structure and thus provide robust adhesion.

Upregulation of KRT19 in the AO of *P. disjunctivus* could be involved in structural stability, helping the organism survive in high-stress environments. KRT19 protein is involved in keratinization, cellular repair, tissue remodelling, and stress responses, as suggested by several researchers [84,86–88]. This aligns with observations in *Xenopus laevis* [89] and *Homo sapiens* [90], where KRT19 plays a pivotal role in preserving tissue integrity and mechanical resilience.

The presence of cytoskeletal proteins such as Myosin heavy chain, Myosin-7, Myosin light chain 13, and Tropomyosin 1 in the AO of *P. disjunctivus*, is interesting. Similar proteins were also identified in skin proteomic studies of *Dicentrarchus labrax* [32] and *Cyclopterus lumpus* [34]. These proteins could help to reinforce tissue integrity and resistance against mechanical stress, as suggested by Hueston et al. [91] and Cole & Welsh [92]. Furthermore, the presence of immune proteins, such as Apolipoprotein A1 and Complement Component 9, in the AO of *P. disjunctivus* is noteworthy. These proteins could support a strong defense against microbial threats. This corroborates reports in *Labeo rohita* [35] and *Cyclopterus lumpus* [33,34], where these proteins were also reported in response to immune defence.

In *P. disjunctivus,* the presence of TBs on the free surface of the AO, could be considered to play significant role in chemoreception. These include the selective identification and sorting of food particles before ingestion [93,94], sensory adaptation, such as gustatory function [95], and sensing of the chemical nature of the surrounding water [25], enabling fish to navigate their surroundings more effectively and with precision. This is crucial for identifying food sources and potential threats [96]. Furthermore, in *P. disjunctivus* AO*,* the location of the TBs on the summit of the mound-like tubercles could be considered an adaptive modification to enhance the efficiency of their sensing ability. Mistri et al. [97], identified TBs at the apical ends of prominent, well-developed epidermal protuberance in *Chaca chaca* as well; and considered these structures a physiological adaptation in relation to sluggish, bottom-dwelling, and predatory lifestyle of the fish. Similar adaptations have also been reported in the epidermis of other fish species. These include *Garra lamta* [23–25], *Labeo rohita*, *Cirrhinus mrigala* [98], and *Hara hara* [18]. Epithelial protuberances equipped with TBs have also been documented on the epithelial surface of the buccal cavity of *Cirrhinus mrigala* [99] and on the epithelial surface of the gill arches of *Rita rita* [95] and have been correlated with their role in the efficient screening of food and enhancement of gustatory function. The widespread occurrence of these features in fish skin and their derivatives highlights their evolutionary significance in aquatic environments.

Collectively, these findings demonstrate that AO significantly contributes to structural, immunological, and environmental adaptability. The diversity in protein composition also reflects an approach of AO to enhance the ability of *P. disjunctivus,* to invade new environments.

## Conclusion

This study provides novel insights into the adaptive structural modifications, including mound-like tubercles separated by deep furrows, spine-like unculi on the surface of these tubercles, MGCs and SGCs secretions, and proteomic analysis of different proteins associated with adhesion in the AO of *P. disjunctivus*. Consequent upon contraction of muscles underlying the AO, mound-like tubercles may be pressed firmly against the substratum. Their relaxation is correlated with the creation of a vacuum, assisting the firm adhesion of the fish to the substratum. Spine-like unculi on the surface of these tubercles help fish to cling to surfaces and scrape food particles while feeding. MGCs and SGCs secreting glycoproteins and proteins respectively, have been considered to function as biological adhesives and lubricants, enhancing adhesion. This observation is supported by proteomic analysis identifying the presence of key adhesion-related proteins (PPL, DSP, and FLNA), which contribute to cell-cell adhesion, structural integrity, and mechanical resilience. Further, identification of proteins involved in keratinization (KRT 8 and KRT 19), cytoskeletal (Myosin-7, Myosin light chain 13, and Tropomyosin 1), and immune defense (Apolipoprotein A1 and Complement Component 9), suggests their involvement in structural integrity and immune defence. TBs are considered to play various functions, e.g., selective identification and sorting of food particles, gustation, and sensing chemical nature and potential threats in the surrounding water. In general, the presence of TBs on the free surface of AO in fish could be viewed as an adaptation that enables them to function more efficiently and with precision. These findings emphasize the multifunctional nature of AO, where structural and molecular adaptations enhance adhesion, stability, and ecological success, emphasizing its evolutionary significance in environments with fast-flowing water. However, the precise mechanism of adhesion to the substratum requires further experimentation. This study highlights the remarkable adaptability of *P. disjunctivus* and provides a foundation for future investigations into the molecular mechanisms of adhesion.

## Supporting information

S1 Table Differentially expressed abundant proteins in ventral skin (control) versus adhesive organ (sample). Differentially expressed proteins identified at a signal-to-noise ratio of 1.5 or above. A Target FDR (strict) of <0.01, |log2FC| > 1, and p-adjusted value ≤ 0.05 were considered. Abbreviation: OS, species; OX, taxonomic identifier; GN, gene name; pI, isoelectric point; MW, molecular weight (kDa). ^a^ Peptide label peptide number sequence matching a protein. ^b^ SC%; sequence score percentage shows the proportion of amino acids in a protein sequence that were found in the sequenced peptides. ^c^ Proteins values considered significant (p-adjusted value ≤ 0.05). ^d^ Fold change is measured as ≤1.
(XLSX)

S2 Table Differentially expressed depleted proteins in ventral skin (control) versus adhesive organ (sample). Differentially expressed proteins were identified using a signal-to-noise ratio of 1.5 or above. A Target FDR (strict) of <0.01, |log2FC| > 1, and p-adjusted value ≤ 0.05 were considered. Abbreviation: OS, species; OX, taxonomic identifier; GN gene name; pI, isoelectric point; MW, molecular weight (kDa). ^a^ Peptide label peptide number sequence matching a protein. ^b^ SC%; sequence score percentage shows the proportion of amino acids in a protein sequence that were found in the sequenced peptides. ^c^ Proteins values considered significant (p-adjusted value ≤ 0.05). ^d^ Fold change is measured as ≤1.
(XLSX)

S3 Table Differentially expressed abundant proteins in dorsal skin (control) versus adhesive organ (sample). Differentially expressed proteins identified using a signal-to-noise ratio of 1.5 or above. A Target FDR (strict) of <0.01, |log2FC| > 1, and p-adjusted value ≤ 0.05 were considered. Abbreviation: OS, species; OX, taxonomic identifier; GN, gene name; pI, isoelectric point; MW, molecular weight (kDa). ^a^ Peptide label peptide number sequence matching a protein. ^b^ SC%; sequence score percentage shows the proportion of amino acids in a protein sequence that were found in the sequenced peptides. ^c^ Proteins values considered significant (p-adjusted value ≤ 0.05). ^d^ Fold change is measured as ≤1.
(XLSX)

S4 Table Differentially expressed depleted proteins in dorsal skin (control) versus adhesive organ (sample). Differentially expressed proteins identified using a signal-to-noise ratio of 1.5 or above. A Target FDR (strict) of <0.01, |log2FC| > 1, and p-adjusted value ≤ 0.05 were considered. Abbreviation: OS, species; OX, taxonomic identifier; GN, gene name; pI, isoelectric point; MW, molecular weight (kDa). ^a^ Peptide label peptide number sequence matching a protein. ^b^ SC%; sequence score percentage shows the proportion of amino acids in a protein sequence that were found in the sequenced peptides. ^c^ Proteins values considered significant (p-adjusted value ≤ 0.05). ^d^ Fold change is measured as ≤1.
(XLSX)

S5 Table List of abundant proteins in the adhesive organ organized in descending order of significance (adjusted p-values ≤ 0.05). Abbreviation: OS, species; OX, taxonomic identifier; GN, gene name. ^a^ Proteins values considered significant (p-adjusted value ≤ 0.05).
(XLSX)

S6 Table List of abundant proteins along with their functions, manually annotated based on data retrieved from UniProtKB and EMBL-EBI databases. (DOCX)

S7 Table Comprehensive details of the abundant proteins used in the STRING protein interaction network. Note: The abbreviations of the protein names used in the protein interaction map are shown in Fig 7b, as assigned by STRING v12.0. Abbreviation: OS, species; OX, taxonomic identifier; GN, gene name. For proteins for which STRING did not provide abbreviations, “NA” has been indicated. ^a^ Human Counterpart UniProtKB Accession Numbers. ^b^ Abbreviations assigned by STRING.
(XLSX)

S1 File Raw SDS-PAGE gel image. Raw image of the SDS-PAGE gel corresponding to Fig 6 (a). Lane 1 – Molecular weight marker (M); Lane 2 – Ventral skin (V); Lane 3 – Adhesive organ (AO); Lane 4 – Dorsal skin (D).
(PDF)
