# Supplementary material for: Nature’s grip: Unveiling the architecture and proteomics of the adhesive organ of a hill stream catfish, Pterygoplichthys disjunctivus
Source: PLoS One. 2025 Oct 9;20(10):e0333933. doi: 10.1371/journal.pone.0333933 (PMC12510517; doi:10.1371/journal.pone.0333933)
Supplement: S6 Table — (DOCX) [file pone.0333933.s006.DOCX]

**S6 Table:** Abundant proteins in the adhesive organ of *Pterygoplichthys disjunctivus* and their functions retrieved manually from UniProtKB and EMBL-EBI databases.

**Note:** Gene ontology (GO) accession numbers of the most abundant adhesive organ proteins are available in S1 and S3 Tables.

| **Proteins having identified GO accession number** | | |
| --- | --- | --- |
| **S.No.** | **Function** | **Protein Name** |
| 1 | Keratinization | Keratin, type II cytoskeletal 8-like; Keratin, type I cytoskeletal 19-like |
| 2 | Adhesion | Calponin-homology domain-containing protein; Desmoplakin, Periplakin; Filamin A-like; Annexin; Tenascin-C; Junction plakoglobin a isoform X1; Fibulin-1 |
| 3 | Cytoskeletal Organization | Myosin-7; Myosin-6-like (fragment); Myosin heavy chain; Myosin, light chain 13; Tropomyosin 1 |
| 4 | Ion Binding | FYVE-type domain-containing protein; Matrilin 1; Cysteine and glycine-rich protein 1; Serotransferrin |
| 5 | ATP Binding | Heat shock protein 90; creatine kinase; ATP synthase subunit beta; Heat shock protein beta-1; ATP synthase subunit beta |
| 6 | Structural molecule activity | Neurofilament medium polypeptide; Uncharacterized protein LOC767716 isoform X1; IF rod domain-containing protein, Peripherin; Clathrin light chain |
| 7 | Oxygen carrier activity | Globin domain-containing protein; Hemoglobin cathodic subunit beta-like |
| 8 | Serine-type endopeptidase inhibitor activity | Inter-alpha-trypsin inhibitor heavy chain H2; Inter-alpha-trypsin inhibitor heavy chain H3 |
| 9 | MAP kinase activity | Mitogen-activated protein kinase |
| 10 | Cytoplasm | Prostaglandin reductase 1; Dihydropyrimidinase-related protein 2 |
| 11 | Protein disulfide isomerase activity | Protein disulfide-isomerase |
| 12 | Peroxiredoxin | Peroxiredoxin-2 |
| 13 | Extracellular region | Complement component C9 |
| 14 | RNA binding | rRNA 2'-O-methyltransferase fibrillarin (Fragment) |
| **Proteins having unidentified GO accession number** | | |
| **S.No.** | **Function** | **Protein Name** |
| 1. | Adhesion | Optineurin, EMAP-like 2, Interstitial collagenase, Ig-like domain-containing protein, LIM zinc-binding domain-containing protein, AHNAK-like protein, Neuroblast differentiation-associated protein |
| 2. | Cytoskeletal Orgainization | Actin-related protein 3, Interstitial collagenase, LIM zinc-binding domain-containing protein, AHNAK-like protein, Myosin heavy chain 7, Neuroblast differentiation-associated protein |
| 3. | Ion binding | Ferritin, EF-hand domain-containing protein |
| 4. | ATP Binding | Myosin heavy chain 7 |
| 5. | Regulation of protease activity | Serpin domain-containing protein |
| 6. | Lipid transport and metabolism | Apolipoprotein A-I |
| 7. | Protein degradation and quality control | Ubiquilin 4 |
| 8. | Glycolysis and cellular metabolism | Glyceraldehyde-3-phosphate dehydrogenase |
| 9. | Gene expression regulation | Transcription factor BTF3 |
|  | | |
