# Supplementary material for: Nature’s grip: Unveiling the architecture and proteomics of the adhesive organ of a hill stream catfish, Pterygoplichthys disjunctivus
Source: PLoS One. 2025 Oct 9;20(10):e0333933. doi: 10.1371/journal.pone.0333933 (PMC12510517; doi:10.1371/journal.pone.0333933)
Supplement: S1 File — Raw image of the SDS-PAGE gel corresponding to Fig 6 (a). Lane 1 – Molecular weight marker (M); Lane 2 – Ventral skin (V); Lane 3 – Adhesive organ (AO); Lane 4 – Dorsal skin (D). (PDF) [file pone.0333933.s008.pdf]

S1_raw_image: Raw image of gel used in the present study

Lane 1 - Molecular marker **(M)**
Lane 2 - Ventral skin **(V)**
Lane 3 - Adhesive organ **(AO)**
Lane 4 - Dorsal skin **(D)**

- Values of molecular weight marker is indicated to the left side, near the ladder.
- The image has been annoted as Fig 6(a), as used in the manuscript’s main figure.
- Image of the gel was captured using a digital camera system (Nikon Coolpix-E5400, Japan) and recorded using an Intel Core i7 computer (Xiaomi, China).

Fig 6 (a)

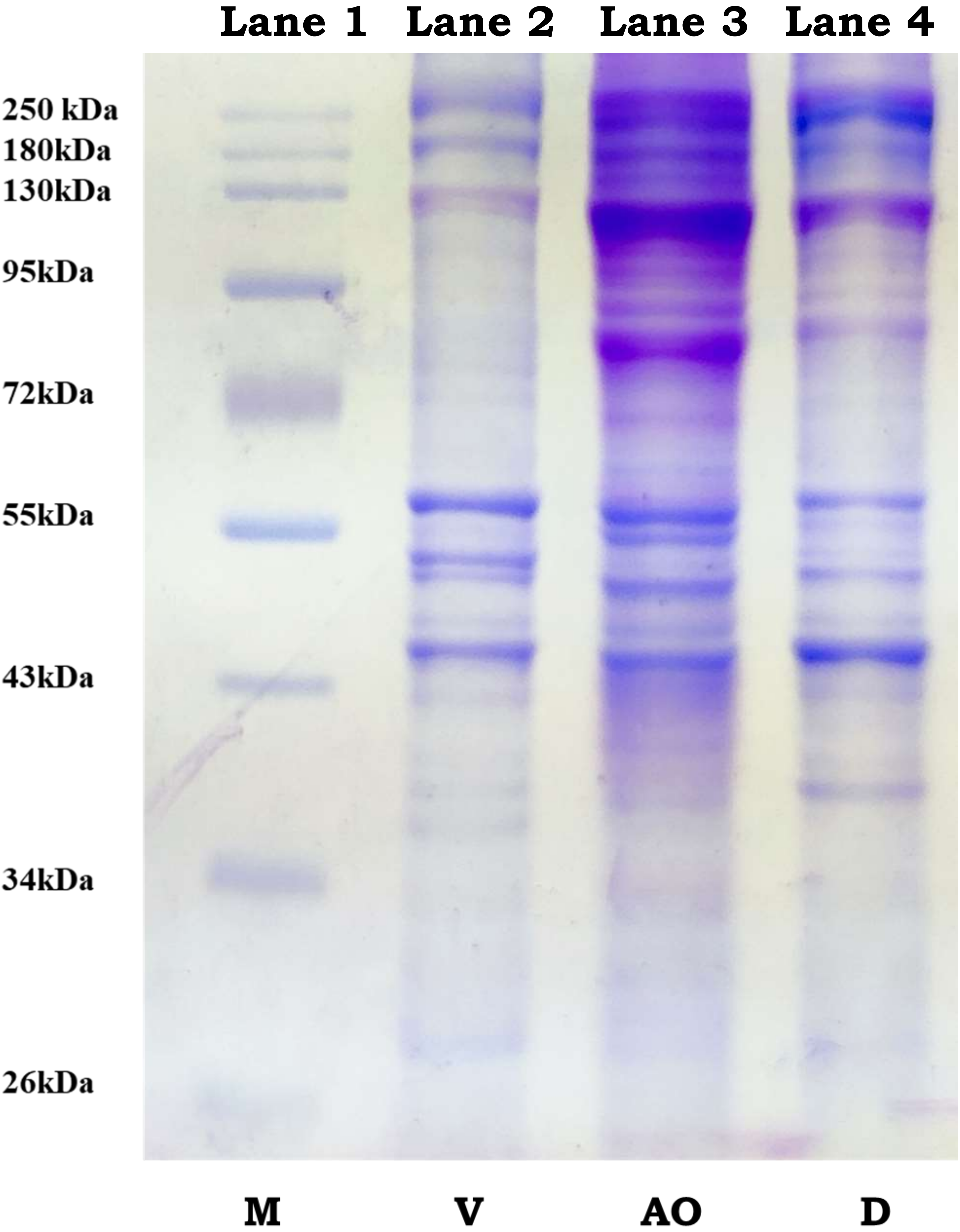
Lane 1
Lane 2
Lane 3
Lane 4
250 kDa
180kDa
130kDa
95kDa
72kDa
55kDa
43kDa
34kDa
26kDa
M
V
AO
D
